# Bullous Melanoma: should the thickness of the bullous lesion be included in Breslow depth measurement?^☆^

**DOI:** 10.1016/j.abd.2021.09.004

**Published:** 2022-01-26

**Authors:** Mariana Abdo de Almeida, Antonio Vitor Martins Priante, Elisangela Manfredini Andraus de Lima, Marcia Lanzoni Alvarenga Lira

**Affiliations:** a Medicine, Universidade de Taubaté, Taubaté, SP, Brazil; b Surgical Clinic, Universidade de Taubaté, Taubaté, SP, Brazil; c Department of Dermatology, Universidade de Taubaté, Taubaté, SP, Brazil; d Department of Pathology, Laboratório do Vale, Taubaté, SP, Brazil

**Keywords:** Dermatopathology, Melanoma, Neoplasia

## Abstract

Bullous melanoma represents a rare variant of melanoma, especially in patients without underlying bullous cutaneous disease. Few cases have been described in the literature, including cases of melanoma in patients with bullous epidermolysis or Hailey-Hailey disease. The histopathological diagnosis of bullous melanoma does not show any difficulties, except for the measurement of the Breslow index. The rarity of this case, the dilemma of how to measure the Breslow index and the importance of an early diagnosis motivated this report.

## Introduction

Bullous melanoma represents a rare variant of melanoma.^1^ The presence of a subepidermal or intraepidermal bullous lesion characterizes the disease.^2^ The rarity of the diagnosis is even greater if the patient does not have an underlying cutaneous bullous disease. Few cases of melanoma in patients with bullous epidermolysis or Hailey-Hailey disease have been described.^3^

The diagnosis of this entity is made using similar criteria (history, dermatological physical examination and anatomopathological analysis) to those used for other variants of the disease; however, the presence of the bullous lesion constitutes a difficulty for the measurement of the Breslow index. The Breslow index constitutes the thickness of the invasive melanoma, measured from the granular layer to the deepest malignant cell, and when there are ulcerated lesions, the measurement from the bottom of the ulceration to the deepest malignant cell is used.^1^

Among the cases already described in the literature, the most commonly identified sites are the heels and feet.^1^

## Case report

**Figure 1:**
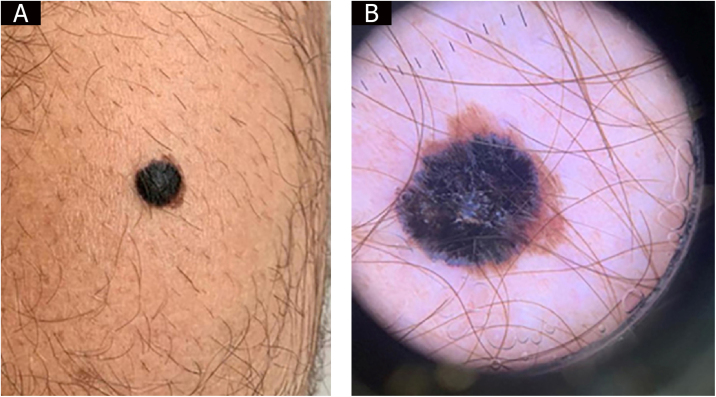
(A), Blackish papule over brownish macula on the left calf. (B), Dermoscopy.

**Figure 2:**
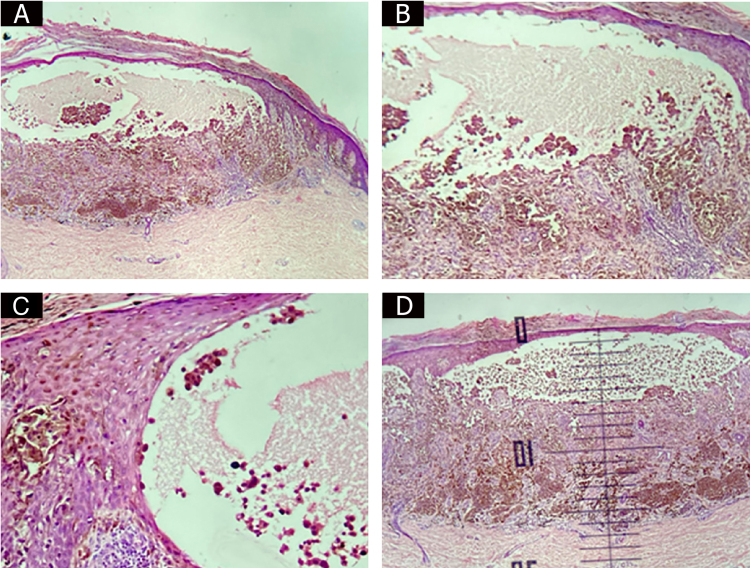
Bullous lesion containing isolated and clustered malignant melanocytic cells.

This is the report of a 44-year-old married male patient who sought dermatological care for a “groin itch for two weeks.” Dermatological examination disclosed the presence of a blackish papule over a brownish macula on the left calf (Fig. 1A). There was no bullous appearance on clinical examination. Dermoscopy showed an atypical brownish pigmented network, with an inverse network surmounted by a homogeneous blackish area with a blue-whitish veil and radiating peripheral streaks, with a clinical diagnostic hypothesis of melanoma or Reed nevus being considered (Fig. 1B). An excisional biopsy was performed, and the anatomopathological examination revealed an invasive melanoma of the superficial spreading type, plus a bullous lesion in the epidermis. The bullous lesion contained isolated and clustered malignant melanocytic cells in addition to serosity (Figs. 2A, 2B, and 2C). There was no ulceration or regression. Surgical margins were free of neoplasia. The Breslow index was measured by subtracting the thickness of the bullous lesion, resulting in 1.1 millimeters (Fig. 2D), as recommended by Woltsche et al.^1^

Sentinel lymph node screening was carried out in the left inguinal region. The immunohistochemistry found no signs of micrometastases. The patient has completed one year and seven months of follow-up without any signs of recurrence.

## Discussion

**Figure 3:**
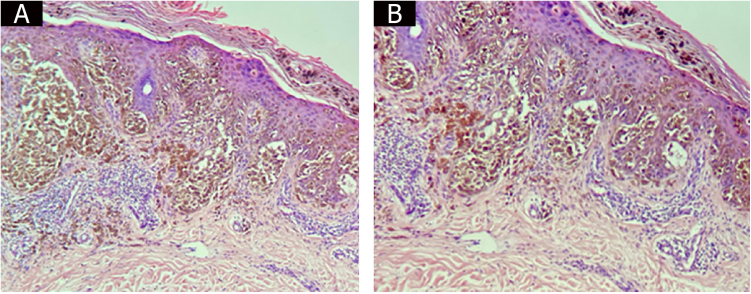
Bullous lesion borders containing tumor cells and loss of intercellular adhesions.

This case report describes a rare variant of bullous lesion-associated melanoma in a patient without a cutaneous bullous dermatosis. There is a debate about the best name to describe melanomas that present with bullous lesions on histopathology. Some terms have already been suggested for the entity. Katorno et al. suggested the term “discohesive melanoma” for cases that show an acantholytic pattern.^4^ Other terms such as “acantholytic-like malignant melanoma” have also been proposed.^5^ The term bullous melanoma is suggested because acantholysis is a term defined as loss of cohesion of desmosomes between epidermal cells^1^ and cannot be a term applied to melanocytes. Moreover, it is evident that the tumor cells start to lose their intercellular adhesions at the borders of the bullous lesion, alterations that are typical of malignant neoplasia (Figs. 3A and 3B). Therefore, terms such as spongiosis or acantholysis are inadequate to describe the mechanism of bullous melanoma formation. The bullous lesion may result from the down-regulation of cadherins, the main adhesion molecule, due to the invasive progression of melanoma cells.^6^ It has also been postulated that in addition to the lack of melanocyte cohesion, friction and microtrauma, as a rare “koebnerization” phenomenon, may be an important factor in the formation of bullous lesions.^7^ However, this would make more sense in lesions found on the feet and heels, which, in fact, are the most common sites, which was not the case in the present patient, in whom the lesion was found on the calf.

A study that analyzed the correlation between cadherin-based adhesion molecules and lesion prognosis showed that higher levels of e-cadherin had a more favorable prognosis in relation to lesions that showed loss of these adhesion molecules.^8^ That said, there is a question here about the prognosis of bullous melanoma: would it have a worse prognosis compared to non-bullous melanoma since the bullous lesion represents a loss of adhesion molecules? The crucial thing, however, is the definition of how to measure the Breslow index in these cases.

Woltsche et al. proposed that the measurement should exclude the thickness of the bullous lesion, as this is not directly related to the tumor mass.^1^ As for Aneiros-Fernandez et al., they chose to include the Breslow index measurement of the bullous lesion in their report.^5^

**Figure 4:**
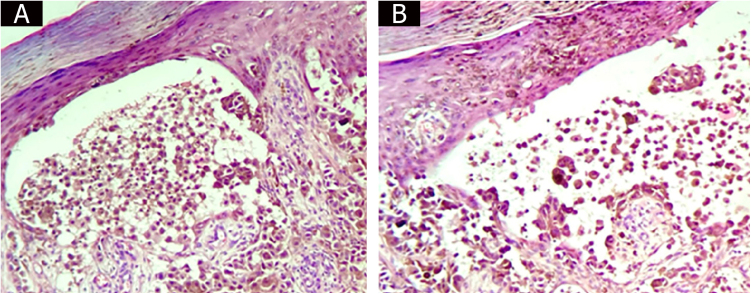
Bullous lesion containing tumor cells.

In the present case, it is evident that the bullous lesion contains tumor cells (Figs. 4A and 4B) and, therefore, it is considered that the bullous melanoma thickness should include the thickness of the bullous lesions. If the thickness of the bullous lesion is disregarded, a large number of malignant cells would be excluded when carrying out the Breslow index measurement. In the present case, to include or not the bullous lesion would not change the pathological staging of the tumor. However, in cases where the measurement of the bullous lesion alters tumor staging, it is thought that the patient would benefit from an “overstaging” rather than an “understaging.”

## Conclusion

The present report describes a case of bullous melanoma, a rare variant of melanoma, mainly in patients with no evidence of a bullous dermatosis. The authors suggest that for bullous melanoma, the Breslow measurement includes the bullous lesion measurement.

## Financial support

None declared.

## Authors’ contributions

Mariana Abdo de Almeida: Statistical analysis; approval of the final version of the manuscript; design and planning of the study; drafting and editing of the manuscript; collection, analysis, and interpretation of data; effective participation in research orientation; intellectual participation in the propaedeutic and/or therapeutic conduct of the studied cases; critical review of the literature; critical review of the manuscript.

Marcia Lanzoni Alvarenga Lira: Statistical analysis; approval of the final version of the manuscript; design and planning of the study; drafting and editing of the manuscript; collection, analysis, and interpretation of data; effective participation in research orientation; intellectual participation in the propaedeutic and/or therapeutic conduct of the studied cases; critical review of the literature; critical review of the manuscript.

Antonio Vitor Martins Priante: Statistical analysis; approval of the final version of the manuscript; design and planning of the study; drafting and editing of the manuscript; collection, analysis, and interpretation of data; effective participation in research orientation; intellectual participation in the propaedeutic and/or therapeutic conduct of the studied cases; critical review of the literature; critical review of the manuscript.

Elisangela Manfredini Andraus de Lima: Statistical analysis; approval of the final version of the manuscript; design and planning of the study; drafting and editing of the manuscript; collection, analysis, and interpretation of data; effective participation in research orientation; intellectual participation in the propaedeutic and/or therapeutic conduct of the studied cases; critical review of the literature; critical review of the manuscript.

## Conflicts of interest

None declared.
